# MutScan: fast detection and visualization of target mutations by scanning FASTQ data

**DOI:** 10.1186/s12859-018-2024-6

**Published:** 2018-01-22

**Authors:** Shifu Chen, Tanxiao Huang, Tiexiang Wen, Hong Li, Mingyan Xu, Jia Gu

**Affiliations:** 1 Shenzhen Institutes of Advanced Technology, Chinese Academy of Sciences, Shenzhen, China; 2 HaploX Biotechnology, Shenzhen, China; 3 University of Chinese Academy of Sciences, Beijing, China

**Keywords:** MutScan, Mutation scan, Variant visualization, Fast detection

## Abstract

**Background:**

Some types of clinical genetic tests, such as cancer testing using circulating tumor DNA (ctDNA), require sensitive detection of known target mutations. However, conventional next-generation sequencing (NGS) data analysis pipelines typically involve different steps of filtering, which may cause miss-detection of key mutations with low frequencies. Variant validation is also indicated for key mutations detected by bioinformatics pipelines. Typically, this process can be executed using alignment visualization tools such as IGV or GenomeBrowse. However, these tools are too heavy and therefore unsuitable for validating mutations in ultra-deep sequencing data.

**Result:**

We developed MutScan to address problems of sensitive detection and efficient validation for target mutations. MutScan involves highly optimized string-searching algorithms, which can scan input FASTQ files to grab all reads that support target mutations. The collected supporting reads for each target mutation will be piled up and visualized using web technologies such as HTML and JavaScript. Algorithms such as rolling hash and bloom filter are applied to accelerate scanning and make MutScan applicable to detect or visualize target mutations in a very fast way.

**Conclusion:**

MutScan is a tool for the detection and visualization of target mutations by only scanning FASTQ raw data directly. Compared to conventional pipelines, this offers a very high performance, executing about 20 times faster, and offering maximal sensitivity since it can grab mutations with even one single supporting read. MutScan visualizes detected mutations by generating interactive pile-ups using web technologies. These can serve to validate target mutations, thus avoiding false positives. Furthermore, MutScan can visualize all mutation records in a VCF file to HTML pages for cloud-friendly VCF validation. MutScan is an open source tool available at GitHub: https://github.com/OpenGene/MutScan

**Electronic supplementary material:**

The online version of this article (10.1186/s12859-018-2024-6) contains supplementary material, which is available to authorized users.

## Background

Next-generation sequencing (NGS) can detect thousands of mutations; however, for some applications, only few of these are targets of interest. For applications such as personalized medicine testing for cancer via NGS technology, clinicians and genetic counselors usually focus on the detection of drugable mutations [1]. For example, both p.L858R mutation and exon 19 deletion of epidermal growth factor receptor (EGFR) gene are highly affected when treating lung cancer patients, since the patients who carry these mutations benefit from EGFR tyrosine kinase inhibitors (TKI) [2]. These mutations can be detected via deep sequencing of patients’ cell-free tumor DNA (ctDNA) [3]. However, the mutated allele frequency (MAF) of variants called in ctDNA sequencing data is very low. Typically, the MAF is usually below 5%, and can even be as low as 0.1% [4]. The need for the detection of mutations with such low MAF drives the development of highly sensitive methods to analyze ctDNA sequencing data [4].

The cconventional mutation detection pipeline for NGS data usually involves different tools for each step. For example, in our regular tumor variant calling pipeline, we use After [5] for data preprocessing, BWA [6] for alignment, Samtools [7] for pipe-up generation, and VarScan2 [8] for variant calling, as well as many auxiliary tools. The different tools used in these steps may cause information loss due to different applied filters, which may finally lead to miss-detection of mutations, especially those with low MAF [9]. This type of false negativity caused by data analysis is not acceptable in clinical applications since it can miss opportunities for better treatment of patients.

In contrast, false positive detection of key mutations should also be avoided since this can introduce expensive but ineffective treatment, and may even cause severe adverse reactions [10]. Conventional NGS pipelines can detect many substitutions and INDELs but unavoidably causes false positives. Particularly, false positive mutations may be detected in the highly repetitive regions of the genome due to inaccurate reference genome mapping of aligners. To reduce this false calling rate, all important mutations must be validated [11]. Variant visualization is a key method for a manual check of mutation confidence. Variant visualizations can be done with tools like IGV [12] and GenomeBrowse, but these tools require slow and inefficient BAM file operations. Especially for visualizing low MAF mutations in ultra-deep sequencing data, IGV or GenomeBrowse is inconvenient since it is difficult to locate the mutated reads among thousands of reads. A fast, lightweight, and cloud-friendly variant visualization tool is therefore needed.

MutScan, the tool presented in this work, is specifically designed to address these problems. It is built on error-tolerant string searching algorithms and is highly optimized for speed with rolling hash [13] and bloom filters [14]. MutScan can run in a reference-free mode to detect target mutations, which are provided via CSV file or pre-defined in the program. With a VCF file and its corresponding reference genome FastA file provided, MutScan can scan all variants in this VCF and visualize them by rendering an HTML page for each variant.

## Implementation

Essentially, MutScan is a highly optimized string-searching program, which scans the input FASTQ files and detects reads that support the mutation targets. In MutScan, a mutation is defined as a combination of (*L, M, R*), in which *M* denotes the mutated bases, *L* denotes the neighbor sequence left to *M* in the reference genome, and *R* denotes the right neighbor sequence. For a read to be considered as a supporting read of a mutation, MutScan requires that a subsequence (substring) of this read exactly matches *M*, and its corresponding left and right neighbor sequences match *L* and *R*, with a few (default is 2) mismatches allowed to support the tolerance of sequencing errors and single-nucleotide polymorphism.

For instance, the EGFR p.L858R mutation locates at chr7: 55,259,515, and the corresponding CDS change is c.2573 T > G. We can extract the context sequence of EGFR p.L858R mutation as:

CATGTCAAGATCACAGATTTTGGGC[G]GGCCAAACTGCTGGGTGCGGAAGAG.

where [G] is the mutation base *M*, CATGTCAAGATCACAGATTTTGGGC is the left neighbor sequence *L*, and GGCCAAACTGCTGGGTGCGGAAGAG is the right neighbor sequence *R*. We denote this whole sequence as the pattern sequence *P*.

For a read sequence *S* to be considered as supporting a mutation, it should be able to align with the pattern sequence *P* of the mutation, and the overlapped region of this alignment should meet the following four conditions:

1. Either *L* + *M* or *M* + *R* is completely covered by overlap (*S*, *P*)
2. Mutation point *M* is exactly matched
3. No insertion or deletion exists around *M* (by default, two left neighbor bases and two right neighbor bases)
4. Edit distance of overlapped sequences should be no more than a threshold *T*_*ed*_ (*T*_*ed*_ = 2 by default)

The reads that meet above conditions will be captured by MutScan, then piled up, and visualized as an HTML page.

### Overall design

**Fig. 1:**
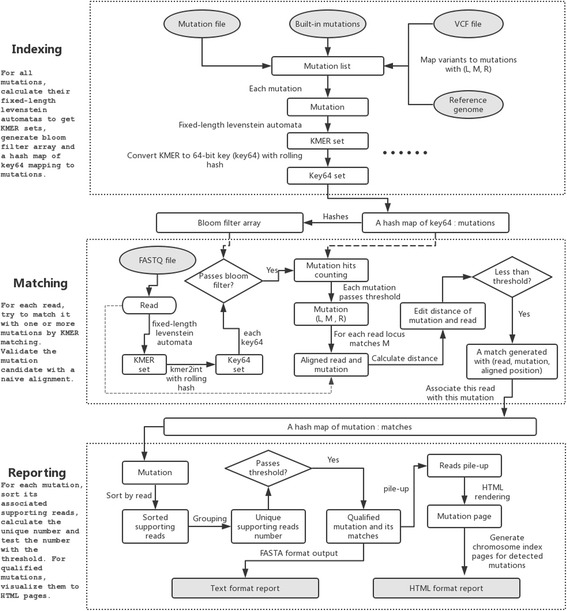
The overall design of MutScan. Three steps are presented: indexing, matching, and reporting. In the indexing step, a hashmap of KMER (all possible substrings of length *k*, *k* = 16 in MutScan’s implementation) mapping to mutations is computed; in the matching step, reads are associated with mutations by looking up the indexed hashmap; in the reporting step, the detected mutations are validated, the supporting reads for each mutation are piled up and rendered to an HTML page. The input and output files are then highlighted in grey

The program flow of MutScan can be divided into three major steps: indexing, matching, and reporting. Figure 1 demonstrates how MutScan works.

In the indexing step, a mutation list is prepared from built-in mutations, a mutation file, or a VCF file. If the input file is a VCF file, the corresponding reference genome of this VCF file should also be provided. A KMER set of each mutation is generated and mapped to 64-bit keys (key64) via hashing functions. An indexing hashmap of key64 mapping to mutations is created.

In the matching step, the KMER set and the corresponding key64 set are computed for each read, and the associated mutations of this read can be found by mapping the key64 set to the mutations using the index computed in the last step. If the key64 mapped count of a mutation is higher than a threshold *T*_*map*_ (*T*_*map*_ = 1 by default), this mutation will be naively aligned with this read. Furthermore, if their edit distance is below threshold *T*_*ed*_ (*T*_*ed*_ = 2 by default), they are considered as matched.

In the reporting step, the supporting reads of each mutation are sorted and a unique number is computed via grouping supporting reads. If the unique number is above a threshold *T*_*unique*_ (*T*_*unique*_ = 2 by default), this mutation is considered valid and will be reported. Then, the reads will be piled up and rendered as an HTML page with the base quality scores represented by colors. Some index pages will also be generated to arrange the mutations by chromosomes, and the HTML format report is finally created.

### Main algorithms

Since the indexing and matching steps are computationally intensive, we use several algorithms to accelerate this process:

1. For all mutations, we first compute their (*L*, *M*, *R*), and then compute the fixed-length Levenshtein automatas [15] of each (*L*, *M*, *R*). *L* and *R* are 20 bp long in MutScan’s implementation. This operation will generate a complete set of target sequences to search.
2. For each sequence in the above set, we use a hash function *kmer2int* to make a 64-bit integer number. A rolling hash [13] method is used to accelerate this process. A hashmap *H* will then be generated, recording every 64-bit key and the mutation list it maps to.
3. A bloom filter [14] is then applied to accelerate the process of checking whether a 64-bit key is in the hashmap *H*. An array *B* of length *N* is initialized with 0, and each key in *H* is hashed to [0, *N*-1] with several hash functions. The value in a position of *B* will be set to 1 if any hash value of a key hits this position. MutScan uses *N* = 2^30^ and uses three different bloom filter hash functions.
4. For each read to scan, we use *kmer2int* to compute the 64-bit integer keys of the read’s KMER. This process is also accelerated via rolling hash.
5. For every 64-bit key computed above, a bloom filter is used to check whether it is in the hashmap *H*. Use the same hash functions in step (3) to obtain the hash values, and check if every value *v* makes *B*[*v*] = 1. If yes, the corresponding sequence is then considered as a potential match, and the mutation list of this key can be obtained from *H*.
6. Every mutation in the above list will be compared to the sequence *S* of this read. With (*L*, *M*, *R*) provided for this mutation, let *P* be the complete pattern sequence (*P* = *L* + *M* + *R*). MutScan first locates the potential mapping of *P* and *S* by finding an exact match of *M* in *S*. We will get the overlapped subsequences of *P* and *S*, which can be denoted as *P*_*O*_ and *S*_*O*_, and can compute their edit distance [16] *d* = *ed.*(*P*_*O*_, *S*_*O*_,). If *d* is not above the threshold *T* (*T* = 2 by default), this read will be added into this mutation’s supporting read set and will be piled with other supporting reads in the visualization process.

### Fixed-length Levenshtein automata

Since the original Levenshtein automata will cause an inconsistent length of the transformed sequences due to insertions or deletions, MutScan computes a fixed-length Levenshtein automata instead of the original. MutScan allows up to one insertion or deletion when searching the mutation patterns from the input reads. To compute the sequence’s Levenshtein automata of fixed-length *F*, MutScan requires an input sequence of a length not shorter than *F* + 2. If an insertion happens in the transformation, the base in the edge will be shifted out, and if a deletion happens, the alternative base outside will be filled in, so the transformed sequences are all of a length *F*.

In our design, only up to two mismatches are allowed by MutScan when searching for the KMER matches between a read and a mutation; therefore, we applied a simplified method to calculate the Levenshtein automata. For a given sequence *S*, its Levenshtein automata is calculated by building a mutated string set of *S* containing all the strings with up to two differences from S, and sampling all the KMER of these strings.

### Rolling hash and kmer2int

The rolling hash used to accelerate the calculation of 64-bit hash resembles the Rabin-Karp string matching algorithm [17]. For a sequence of length *k*, this hash function maps a sequence to an integer as follows:

$$ H={b}_1{a}^{k-1}+{b}_2{a}^{k-2}+{b}_3{a}^{k-3}+\dots \cdots +{b}_k{a}^0 $$

where *a* represents a constant number, *b*_*i*_ represents the number representing the base at position *i*. We use *a* = 2 since *k* is usually above 40, and the hash value can be greater than 2^64^ if we use *a* = 3 or above. It is difficult to choose the values representing different bases (A/T/C/G/N), too small or too simple values will cause heavy hash collisions. After many iterations, the following odd numbers were chosen: A = 517, *T* = 433, C = 1123, G = 127, *N* = 1.

This hash function can be used to accelerate the hash calculation of Levenshtein automatas or subsequences of reads. For example, if we already have a sequence *S*_*1*_ and its hash value *H*(*S*1); then, we transform *S*_*1*_ to *S*_*2*_ by replacing the 5th base A by T and the hash value of *S*_*2*_ can be computed as:

$$ H\left({S}_2\right)=H\left({S}_1\right)-{2}^{k-5}\times 517+{2}^{k-5}\times 433 $$

When we compute all hashes of a sequence S, we can compute the hashes one by one by sliding the fixed-width window over S. Except for the first hash that is fully computed, the remaining hashes can be computed quickly. For example, let *H*(*S*_1…*k*_) denote the hash of the 1…k sub-sequence of the sequence S. When the needle moves to the next window, the hash of the 2…k + 1 sub-sequence *H*(*S*_2…*k* + 1_) can be computed as:

$$ H\left({S}_{2\dots k+1}\right)=\left(H\left({S}_{1\dots k}\right)-{b}_1{2}^{k-1}\right)\times 2+{b}_{k+1} $$

This calculation can be very fast since the multiplication with 2 or 2^k-**1**^ can be conducted by bit shifting operations.

### Bloom filter

When testing a read to find whether it matches any of our target sequences, we should compute its hashes and compare them to the pre-computed Levenshtein automata set. Since this function will be very heavily used, it should be optimized to avoid performance bottlenecks. The Levenshtein automata set is stored as a hashmap, whose keys are 64-bit integers. The keys are sorted, and by default, a binary search method is applied to find whether a given key is in the key set of the hashmap, which is usually not efficient. It requires about 28 comparisons to test a key against a key set with 256 elements.

Bloom filter can be applied to accelerate the hit-or-not testing of a key against a key set. As explained above, an array *B* of length *N* is initialized with 0 at each position, and hash values of multiple hash functions for each 64-bit key are computed. The element of *B* is set to 1 if any hash value of any key hits the particular position. When testing a given key *K*, compute its hash values of the same hash functions. *K* is not a member of the key set if any hash value hits 0. The trick is that most keys are not members of the key set so that the testing will return false after a few checks (typically one or two).

MutScan uses three different hash functions to map 64-bit integers to [0, 2^30^-1], all of which have the following form:

$$ {f}_i(key)=\left({\mathrm{v}}_{\mathrm{i}}\times \mathrm{key}\right)\&\left({2}^{30}-1\right),\kern1em i=1,2,3 $$

Large odd numbers can be chosen for *v*_*i*_, MutScan uses *v*_*1*_ = 1,713,137,323, *v*_*2*_ = 371,371,377, and *v*_*3*_ = 7,341,234,131.

### Paired-end read merging

Sequence length is also a factor that affects mutation detection. To obtain a longer sequence, MutScan tries to merge each pair of reads for paired-end sequencing data. For a read pair *R*_1_ and *R*_2_, *rcR*_2_ is computed as the reverse complement of *R*_2_. The merging algorithm searches the biggest overlap of *R*_1_ and *rcR*_2_, while their overlapped subsequences are entirely identical. If the overlapped region is longer than a threshold (by default, *T*_len_ = 30 bp), we consider them as overlapped and merge them to a single read. We can obtain longer sequences after merging read pairs, and continue the matching process even if the mutation point locates on the edge of reads. If one pair of reads cannot be merged, MutScan will process them. Although a sequencing library with large insert sizes would prohibit the overlap of read pairs, it will not cause too much impact on performance since MutScan can even work well for single-end sequencing data.

### Visualization

**Fig. 2:**
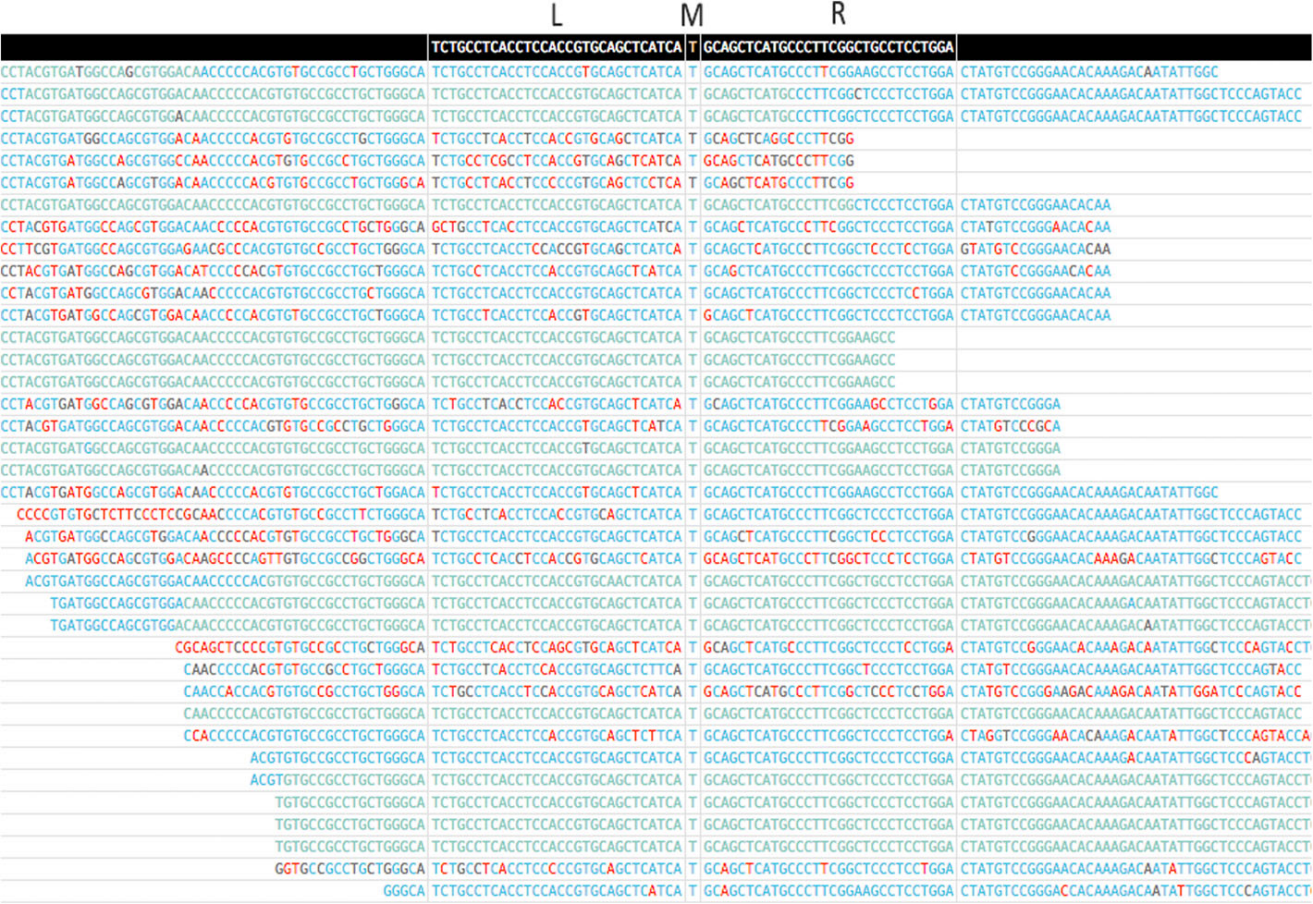
Screenshot of a MutScan’s pile-up result. The demonstrated mutation is EGFR p.T790 M (hg19 chr7:55,249,071 C > T), which is an important drugable target for lung cancer. This mutation’s (*L*, *M*, *R*) sequences are provided at the top of this figure, and *M* is the mutation base (C > T). The color of the bases indicates the quality score (green and blue indicate high quality, red indicates low quality). This screenshot is incomplete, and the complete report can be found at http://opengene.org/MutScan/report.html

In the visualization stage, MutScan generates an HTML file for each mutation, in which all supporting reads are piled up. MutScan cuts each supporting read by its mapping to (*L*, *M*, *R*) sequences of its mutation. MutScan sorts each mutation’s supporting reads by its starting and ending positions and considers the supporting reads with identical positions as duplicates of one unique read. Bases on the HTML page are colorized according to their quality scores. Figure 2 provides an example of a mutation’s pile-up HTML graph.

Another advantage of MutScan is that it can visualize read duplications. Since the supporting reads of each mutation are sorted by their coordination, reads sharing the same coordination will be grouped. Multiple reads that share the same coordination will be displayed as a block and thus can easily be found from the visualization result. Such a block of reads can be considered as a single unique read. In the HTML report of MutScan, both numbers of supporting reads and unique supporting reads are given for each mutation.

## Results and discussion

MutScan can be used to both detect and visualize target mutations. For example, in clinical genetic testing for cancer, several hotspot mutations are highly concerned by oncologists. MutScan contains a built-in list with most actionable gene mutations for cancer diagnosis [18]. It can scan these pre-defined mutations in a very fast way, which is typically at least 20X faster than conventional complete pipelines.

The visualization functions make MutScan applicable for variant validation. MutScan generates an HTML page for each mutation with its supporting reads piled up, from which users can evaluate the confidence of a mutation by calculating the supporting read number, the quality scores of the bases at a mutation point, the rate of duplication, and the form of overlapping read pairs.

### Sensitivity and specificity

A major concern is MutScan’s mutation detection sensitivity for key mutations. Since MutScan was initially developed for tumor mutation detection and visualization, we conducted an experiment to evaluate the mutation sensitivity using 28 mutation-positive tumor samples. These samples were either circulating tumor DNA samples (ctDNA) or formalin-fixed, paraffin-embedded (FFPE) tissue samples. The DNA samples extracted from these were target captured using a cancer targeting sequencing panel with a size of around 100 K base pairs. The sequencing depth was at least 5,000X for ctDNA, and 1000X for FFPE samples to detect mutations with low MAF. In this evaluation, we focused on seven actionable oncogene mutations of four genes, which are p.T790 M/p.E746_A750delELREA of the EGFR gene, p.V600E of the BRAF gene, p.H1047R/p.E545 K/p.E542K of the PIK3CA gene, and p.G12D of the KRAS gene. Among these seven mutations, six are a single nucleotide variation (SNV), and the remaining mutation is a 15-bp deletion [19–21]. All 28 samples underwent at least one of these 10 mutations, and some samples underwent two or more mutations.

**Fig. 3:**
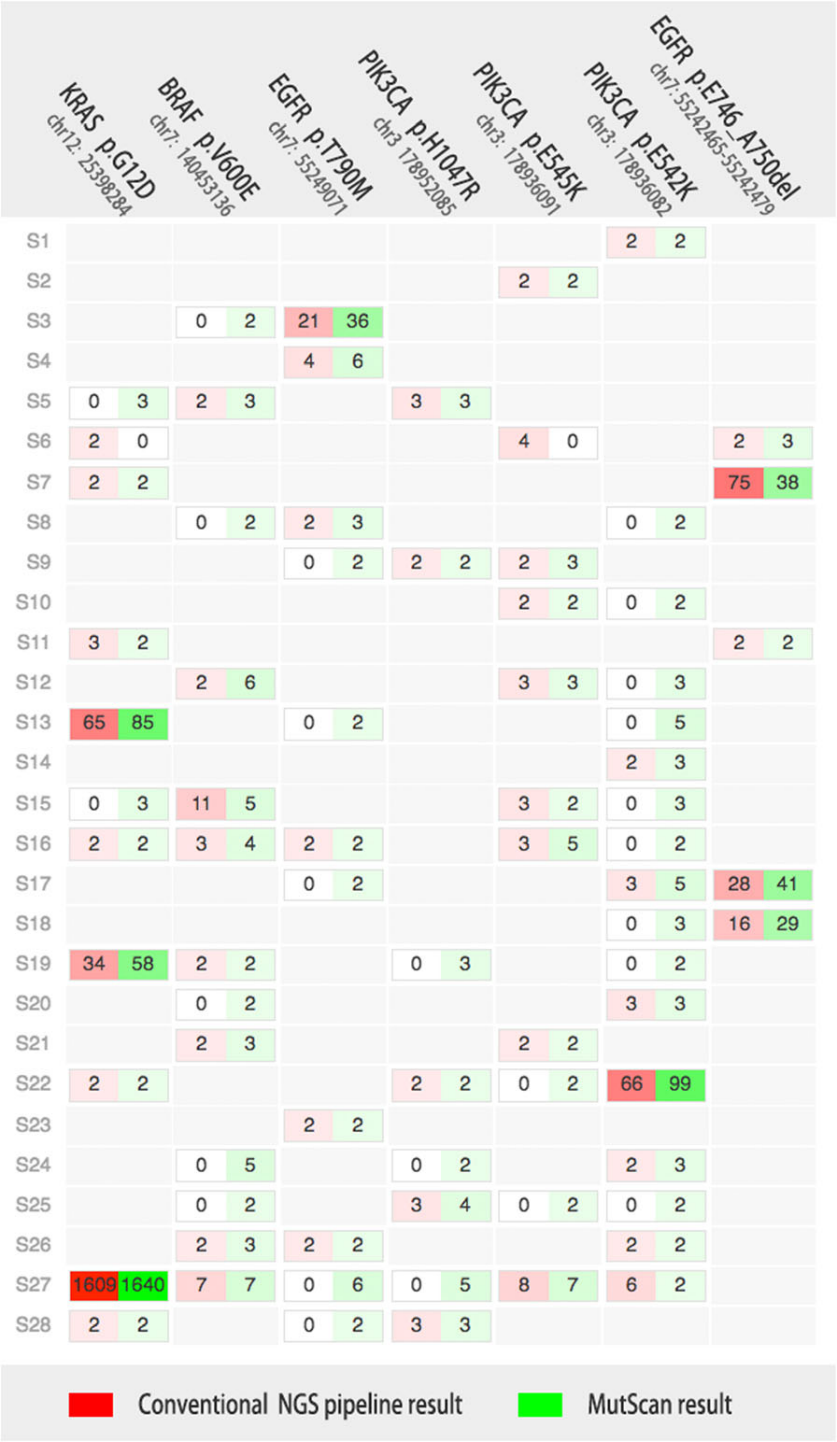
Comparison result of MutScan and conventional NGS pipeline. The conventional NGS is a tumor variant calling pipeline using AfterQC + BWA + Samtools + VarScan2, which can be found at https://github.com/sfchen/tumor-pipeline. Mutations are given in columns and samples are given in rows. Tumor pipeline detected mutations are highlighted in shades of red, and MutScan detected mutations are highlighted in shades of green. The depth of the color reflects the unique supporting read number, which is also shown in the table cells

In this evaluation, MutScan was compared to a tumor variant calling pipeline of (AfterQC + BWA + Samtools + VarScan2), which can be found at GitHub (http://github.com/sfchen/tumor-pipeline). The unique supporting read number for each mutation was computed via MutScan and the tumor pipeline respectively, and the result of the comparison is shown in Fig. 3.

Figure 3 shows that most mutations detected by the tumor pipeline were also detected by MutScan, except for the S6 sample, which was reported with two unique reads of KRAS p.G12D and three unique reads of PIK3CA p.E545 K detected by tumor pipeline, but not detected by MutScan. By manually checking the data of this sample, we found that the miss-detection of these two mutations was caused by too many mismatches of their supporting reads. In most cases, the unique supporting read numbers of tumor pipeline detected mutations and MutScan detected mutations were close. Furthermore, MutScan also detected some mutations with less than six unique supporting reads that were not detected by tumor pipeline, suggesting that MutScan is more sensitive than tumor pipeline for the detection of these key mutations. We checked the reads of all mutations that were only detected by MutScan and found that almost all reads perfectly matched their corresponding mutations, indicating that MutScan’s read searching algorithm rarely made false calling for these target mutations. Considering that only one unique supporting read is not confident to call a variant, MutScan provides an option to set the minimum unique supporting reads (2 as default) to obtain high specificity.

### Speed and memory usage evaluation

To evaluate the speed of MutScan, we compared its running time against two conventional pipelines; one is the tumor pipeline of AfterQC + BWA + Samtools + VarScan2, the other is GATK best practices pipeline with GATK HaplotypeCaller [22]. Since we did not evaluate tumor/normal paired samples, we did not use the MuTect2 pipeline [23], which is even slower than GATK HaplotypeCaller. We first ran these two pipelines to generate the respective VCF files, and then passed these files to MutScan for detection and visualization. We also ran MutScan with built-in actionable mutations. Eight pairs of paired-end sequencing FASTQ files were used for this speed evaluation, the execution times of all tests were recorded, and the comparison result is shown in Table 1.

**Table 1:** Execution time comparison of MutScan and conventional pipelines

Sample ID	Base number	GATK pipeline called variants	Tumor pipeline called variants	GATK pipeline time	Tumor pipeline time	MutScan time (GATK VCF)	MutScan time (tumor pipeline VCF)	MutScan time (built-in mutation)
S001	3.07 G	376	1163	166m01s	84m26s	3m09s	4m31s	1m28s
S002	2.70 G	376	1438	158m40s	64m09s	3m03s	4m50s	1m11s
S003	4.98 G	531	949	236m57s	135m47s	4m57s	6m59s	2m19s
S004	3.51 G	375	798	186m48s	100m14s	3m16s	4m26s	1 m 34 s
S005	3.50 G	385	751	191m29s	84m24s	3m26s	4m20s	1m34s
S006	3.67 G	359	1303	182m42s	96m50s	3m24s	5m57s	1m36s
S007	6.08 G	380	2055	200m22s	142m30s	4m20s	11m17s	2m29s
S008	3.33 G	383	873	175m16s	90m27s	2m52s	4m37s	1m20s

The input files are Gzip compressed paired-end sequencing FASTQ, and the base number is the summation of both paired files. Since the tumor and GATK pipeline used different variant detection and filtering strategies, the tumor pipeline detected more variants than the GATK pipeline. The column MutScan (GATK VCF) is the execution time of MutScan for processing time with the VCF (INDEL + SNV) called by the GATK pipeline, similarly for the column MutScan (tumor pipeline VCF). When MutScan was ran with a VCF, its execution time was predominantly determined by the size of the FASTQ file and the number of variants

In this evaluation, BWA alignment was both involved in the GATK pipeline and the tumor pipeline, and both pipelines used 10 threads for running BWA. GATK HaplotypeCaller of the GATK pipeline and VarScan2 of tumor pipeline were run with a single thread. We found the alignment (BWA) and the variant calling (GATK/VarScan2) components took more than 80% of the total running time. MutScan was run with a single thread for indexing, four threads for matching, and a single thread for reporting. Despite having the difference of threading settings, we still found that MutScan is much faster than both the GATK pipeline and the tumor pipeline.

During this evaluation, we also found that MutScan could eliminate some false positives called by conventional pipelines. For example, we found some mutations near chr22: 42,526,561 appeared in all the eight samples and surmised that they should be false positives. By manually looking into the alignment, we found about seven mismatches near that genome position, and confirmed that those mutations were false positives caused by bad alignment. For details of the GenomeBrowse software, please refer to Additional file 1. Since MutScan does not call variants with too many mismatches, these mutations will not be detected.

When processing large FASTQ files with many target mutations, MutScan may use too much memory since it keeps all the supporting reads in RAM. To address such problem, MutScan provides a simplified mode to reduce the memory usage. In the simplified mode, each supporting read’s quality string and the name string are not kept, and the sequence string is compressed as a 2-bit buffer. The four bases (A, T, C, G) are represented by two bits (00, 01, 10, 11), and the read contains N bases will be discarded since it usually indicates low quality and N base cannot be represented in the 2-bit format. The simplified mode also requires less mismatches when comparing the reads and the target mutations, consequently it’s faster than the normal mode since the Levenshtein automata is smaller. We conducted an experiment to evaluate the time and memory used by MutScan for processing FASTQs and mutations in different sizes, and the result is shown in Table 2.

**Table 2:** Time (in second) and memory (in megabytes) used by MutScan for processing FASTQs and mutations in different sizes

Mutation Number➔	5 K Mutations		10 K Mutations		50 K Mutations	
MutScan Mode➔	Simplified	Normal	Simplified	Normal	Simplified	Normal
5Gbp FASTQ	255 s, 672 M	370 s, 1542 M	289 s, 683 M	428 s, 2110 M	380 s, 943 M	537 s, 9113 M
10Gbp FASTQ	402 s, 692 M	621 s, 2260 M	447 s, 714 M	648 s, 3201 M	624 s, 1113 M	2279 s, 15,569 M
50Gbp FASTQ	1622s, 769 M	2956 s, 8352 M	1897s, 929 M	3469 s, 12,440 M	2729 s, 2601 M	10,927 s, 69,389 M

The input was paired-end data, and the base number was the sum of read1 and read2. Both the simplified mode and the normal mode were evaluated and shown in the table

From Table 2, we can learn that the memory usage is nearly linear related to the size of FASTQ and the number of mutations. The running time is also nearly linear related to the size of FASTQ, but is not linearly related to the number of mutations. Much less memory and running time is used in the simplified mode than the normal mode. As a side effect, a small part of the supporting reads with many sequencing errors or some N bases cannot be detected in the simplified mode since it applies stricter comparison strategy. In our evaluation, about 1% reads that were detected in the normal mode couldn’t be detected in the simplified mode, which is still acceptable in most cases. The quality string is not kept in the simplified mode so the quality score of each base is not available in the report. The simplified mode can be explicitly enabled or disabled by the command line arguments. By default, Muscat will evaluate the FASTQ file size and automatically enable the simplified mode if the evaluated FASTQ file size is larger than 50GB and the mutation number is more than 10,000.

### Limitations and future work

Although MutScan can detect and visualize target mutations in a fast way, it still has some disadvantages and can be improved in future versions.

The first major disadvantage is that if the target mutation is coupled with a long insertion or deletion in its neighbor sequence, MutScan may fail to detect it. In the speed evaluation using eight samples, a total of 2646 single-nucleotide polymorphism (SNPs) were called by the GATK pipeline, all the SNPs were detected by MutScan with VCF input except three mutations, and all of these undetected mutations were coupled with long INDELs. For example, one of these three mutations is a C > T substitution located at chr4: 55,146,389 (dbSNP id = rs1547904), and it has a coupled T > TTGTAGGTCCCCCAG insertion located at chr4: 55,146,406 (dbSNP id = rs6148442), which is very close to the target mutation. To address this issue, the neighbor variants (i.e., SNPs) should be considered when searching for matches, which will be implemented in future.

Another significant disadvantage is that MutScan only supports substitutions and INDELs, while it does not support gene fusions. Gene fusions are also important for cancer genomics, and there are also a lot of drugable gene fusions. For example, a patient with a fusion of the echinoderm microtubule-associated protein-like 4 (EML4) gene and the anaplastic lymphoma kinase (ALK) gene can be treated with Crizotinib. MutScan has difficulties catching these gene fusions since the breakpoints of these fusions are usually varying, and consequently, we cannot generate MutScan compatible mutations with (*L*, *M*, *R*). The authors have partially addressed this problem by creating a gene fusion detection and visualization tool, which is called GeneFuse and is also an open source project (https://github.com/OpenGene/GeneFuse).

## Conclusion

In clinical applications, it is essential to seek low MAF drugable mutations from ultra-deep sequencing data. In contrast to traditional variant discovery strategies (filtering, mapping, deduplicating, and variant calling), MutScan provides a novel way to directly detect target mutations from raw FASTQ files. Since it is based on direct error-tolerant string searching algorithms, MutScan can achieve very high sensitivity. Furthermore, MutScan can visualize variants by generating HTML-based read pile-ups, and consequently provide a cloud-friendly method to validate variants called by conventional pipelines.

In summary, MutScan is a fast, standalone and lightweight tool aimed to detect target mutations from raw FASTQ data or to validate mutations by generating HTML-based read pile-up visualizations.

## Additional file

Additional file 1: A screenshot of false positive caused by bad alignment at chr22. We found some mutations near chr22:42,526,561 appeared in all the eight samples and surmised that they should be false positives. By manually looking into the alignment, we found about seven mismatches near that genome position, and confirmed that those mutations were false positives caused by bad alignment. (PNG 193 kb)

## Abbreviations

ALK: Anaplastic lymphoma kinase
BRAF: B-Raf proto-oncogene, serine/threonine kinase
ctDNA: Circulating tumor DNA
EGFR: Epidermal growth factor receptor
EML4: Echinoderm microtubule-associated protein-like 4
FFPE: Formalin-fixed, paraffin-embedded
IGV: Integrative genome viewer
INDEL: Insertion and deletion
KMER: All possible substrings of length k
KRAS: Proto-oncogene corresponding to the oncogene first identified in Kirsten rat sarcoma virus
MAF: Mutated allele frequency
NGS: Next-generation sequencing
PIK3CA: Phosphatidylinositol-4,5-bisphosphate 3-kinase catalytic subunit alpha
SNP: Single-nucleotide polymorphism
SNV: Single-nucleotide variant
