# Supplementary figures and images for: MutScan: fast detection and visualization of target mutations by scanning FASTQ data
Source: BMC Bioinformatics. 2018 Jan 22;19:16. doi: 10.1186/s12859-018-2024-6 (PMC5778627; doi:10.1186/s12859-018-2024-6)

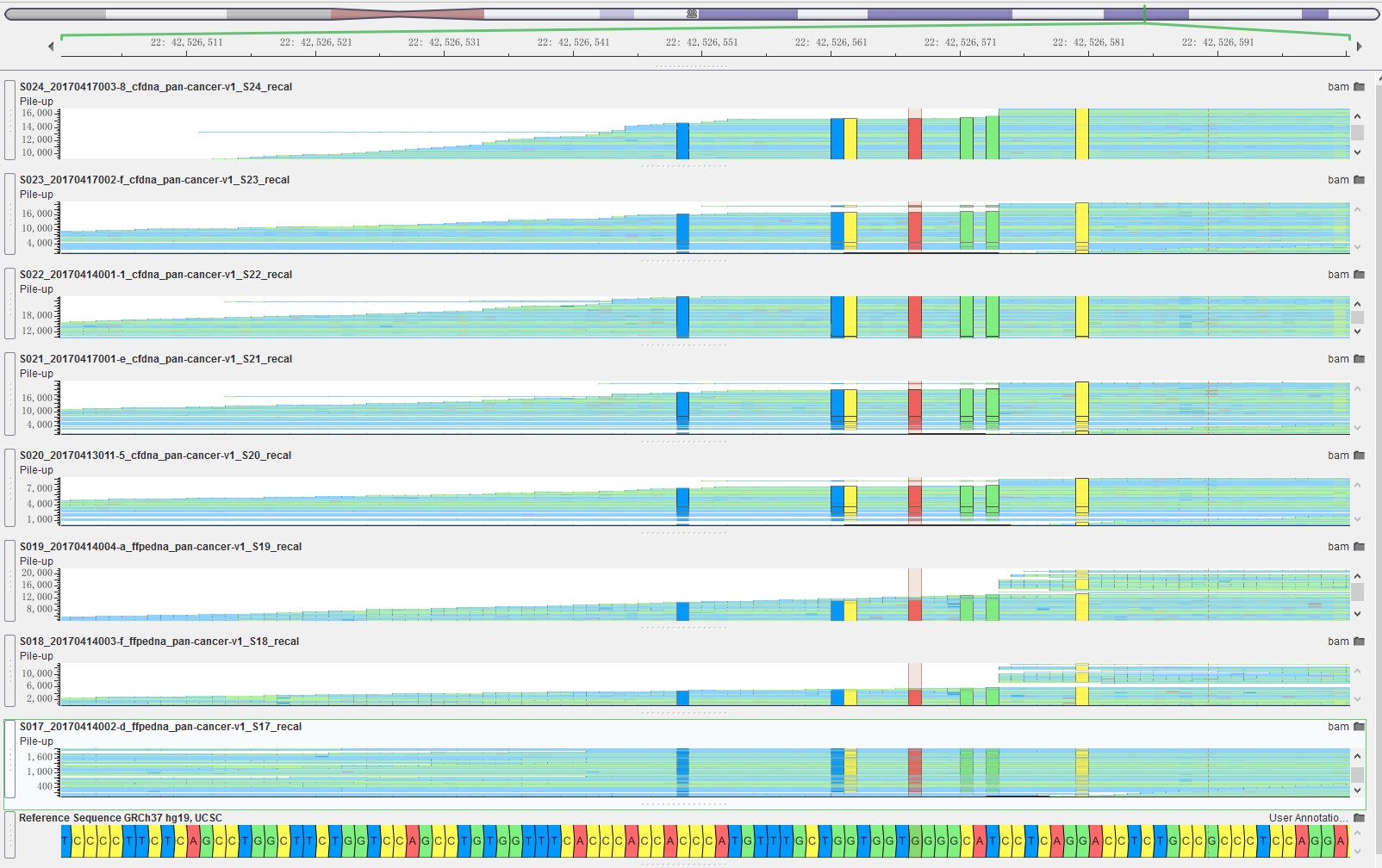

Supplement: Additional file 1: — A screenshot of false positive caused by bad alignment at chr22. We found some mutations near chr22:42,526,561 appeared in all the eight samples and surmised that they should be false positives. By manually looking into the alignment, we found about seven mismatches near that genome position, and confirmed that those mutations were false positives caused by bad alignment. (PNG 193 kb) [file 12859_2018_2024_MOESM1_ESM.png]
